# Determination of Carrageenan in Livestock and Poultry Meat by Ultrahigh-Performance Liquid Chromatography-Tandem Mass Spectrometry

**DOI:** 10.1155/2021/5277453

**Published:** 2021-09-24

**Authors:** Yanqin Sun, Xudong Zhu, Xixi Shen, Wei Wang

**Affiliations:** ^1^Key Laboratory of Meat Processing and Quality Control, MOE, Key Laboratory of Meat Processing, MARA, Jiangsu Synergetic Innovation Center of Meat Processing and Quality Control, Nanjing Agricultural University, Nanjing 210095, China; ^2^College of Sciences, Nanjing Agricultural University, Nanjing 210095, China

## Abstract

Ultrahigh-performance liquid chromatography-tandem mass spectrometry (UHPLC-MS/MS) has become the main method for the detection and analysis of food additives because of its good separation, high selectivity, and high sensitivity. The aim of this study was to establish an UHPLC-MS/MS method that can quickly and accurately measure the content of carrageenan in livestock and poultry meat. Chromatographic separation was performed on an ACQUITY UPLC BEH HILIC C18 column (2.1 mm × 50 mm, 1.7 *μ*m) using a gradient elution with methanol and 0.1% (v/v) formic acid in water as a mobile phase. The quantitative analysis was executed using a triple quadrupole mass spectrometer in which electrospray ionization, multiple reaction monitoring, and negative mode were operated. The retention time was about 1.3 min for carrageenan. The carrageenan content showed a good linear relationship from 0.05 to 1.00 g/kg. The limit of detection (LOD) was 0.06 g/kg, and the limit of quantification (LOQ) was 0.18 g/kg. The standards were spiked at three levels (low, medium, and high) and were analyzed in six replicates. The recovery values of carrageenan in pork, beef, lamb, chicken, and duck meat were 82.06–111.55%, 85.43–112.50%, 89.55–116.00%, 83.80–102.15%, and 82.41–110.90%, respectively. The relative standard deviations (RSDs) were all lower than 7.51%. The developed method shows a high recovery rate and good precision and can be used for the rapid detection of carrageenan in livestock and poultry meat.

## 1. Introduction

Carrageenan is a polysaccharide extracted from marine red algae and widely used in the production and processing of meat and meat-based products to improve meat texture [1–3] because of its good water retention capacity and stability [4–6]. Carrageenan is a safe and nontoxic natural food additive. The carrageenan added to meat and meat-based products is nondegradable. Even this nondegradable type, however, can be degraded under high temperature and an acidic environment during meat processing [7–9]. According to the references and results of animal experiments, excessive intake of degraded carrageenan damages the gastrointestinal tract, induces inflammation, and leads to the development of tumors and other diseases [10–14]. In this regard, the safety of carrageenan in meat and meat-based products should be carefully regulated.

The European Union currently prohibits the addition of carrageenan to infant food formulas but allows its use in meat and meat-based products. The *National Food Safety Standard for the Use of Food Additives* (GB2760-2014) issued by China clearly indicates that carrageenan cannot be used as a thickening agent in fresh meat, but it can be used in the production of premade meat products and cooked meat products with clear marking on the product packaging. Carrageenan is not strictly regulated in the current marketplace, but driven by profits, some unethical manufacturers use carrageenan to increase the weight of meat and meat-based products. The unregulated use of carrageenan not only changes the composition of meat but also increases the risk of contamination from foreign impurities and pathogenic microorganisms. This seriously affects meat quality and safety [14]. In addition, carrageenan is often used as an adhesive to reconstitute meat to maintain its sensory state. To attract consumers, manufacturers do not always correctly or accurately label the food packaging as required by government standards. Thus, it is important to detect the presence of carrageenan in livestock and poultry meat and related products.

Current carrageenan detection methods in complex food products mainly include fluorescence spectroscopy [15, 16], infrared spectroscopy [17–21], Raman spectroscopy [22], and nuclear magnetic resonance technology [23–26]. These methods all require models established in conjunction with chemometrics, and data processing is cumbersome. High-performance liquid chromatography-tandem mass spectrometry (HPLC-MS/MS) combined with chromatographic separation and mass spectrometry identification can help determine a variety of compounds—this technique has been widely used in the analysis of food quality and safety including for aflatoxin [27], free fatty acids [28], and phenols [29]. However, there are few reports on the detection of carrageenan in livestock and poultry meat via this method. Ultrahigh-performance liquid chromatography-tandem mass spectrometry (UHPLC-MS/MS) has even higher separation speed, better peak identification, and higher sensitivity than HPLC-MS/MS. Therefore, in view of the fact that carrageenan is mainly artificially added and does not exist in fresh meat, we established a UHPLC-MS/MS method to quickly measure the content of carrageenan in livestock and poultry meat. This method can realize sensitive and quantitative identification of the carrageenan present in livestock and poultry meat and detect carrageenan-adulterated meat and poultry.

## 2. Materials and Methods

### 2.1. Materials and Reagents

Food-grade carrageenans were purchased from ShiShi Globe Agar Industries Co., Ltd. (Fujian, China). Hydrochloric acid (density approximately 1.19 g/mL, guaranteed reagent grade) was obtained from Nanjing Chemical Reagent Co., Ltd. (Nanjing, China). Chromatographically pure acetonitrile was purchased from Merck Co., Ltd. (Branchburg, NJ, USA). All solutions were prepared with ultrapure water (18.2 MΩ cm, an Arium® Pro ultrapure water purification system; Sartorius, Göttingen, Germany). We obtained positive samples of pork, beef, lamb, chicken, and duck containing 0.20 g/kg carrageenan labeled as A, B, C, D, and E, respectively. Samples were from the Supervision, Inspection and Testing Center for Quality of Meat Products (Nanjing, China). In addition, we acquired commercial food products including various kinds of livestock and poultry meat and meat products from a local supermarket in Nanjing, China. Other analytically pure reagents were obtained from Nanjing Chemical Reagent Co., Ltd. (Nanjing, China).

### 2.2. Sample Pretreatment

Food-grade carrageenans have an average molecular weight of 100,000 Da or higher [24], which makes it difficult to detect them directly using UHPLC-MS/MS. Therefore, in this experiment, we pretreated the meat samples according to the following steps: (1) about 10.00 g of meat sample was placed in a 50 mL centrifuge tube followed by the addition of 23 mL of water; (2) the mixture was homogenized at 10,000 rpm for 1 min, and then 2 mL of 12 mol/L hydrochloric acid was added and the mixture was vortexed for 1 min; (3) the sample was placed on a shaker at 200 rpm for 30 min; (4) the mixture was then put in a water bath at 80°C for 20 min and agitated every 10 min; (5) the sample was taken out from the water bath and cooled to room temperature (22°C–28°C) before centrifugation at 10,000 rpm for 10 min; and (6) the supernatant was diluted with acetonitrile at a ratio of 1 : 1 (v/v) and passed through a 0.22-*μ*m filter into a UHPLC-MS/MS autosampler vial.

### 2.3. Preparation of Standard Solutions

In a 10 mL volumetric flask, 0.10 g of carrageenan was dissolved in 8 mL of water at 60°C. The flask was cooled to room temperature (22°C–28°C) before further dilution with water to prepare a stock solution with a mass concentration of 10 mg/mL. We weighed 10.00 g of five types of livestock and poultry meat without carrageenan into a 50 mL centrifuge tube. We then added 0.05, 0.10, 0.20, 0.40, 0.60, 0.80, or 1.00 mL of carrageenan reference stock solution to the tubes along with water to reach a final volume of 23 mL. The mixture was homogenized for 1 min at 10,000 rpm. The subsequent pretreatment was performed in accordance with the method described above, and the extracts were analyzed through UHPLC-MS/MS.

### 2.4. Chromatographic and Mass Spectrometric Conditions

We used an ACQUITY I-Class UHPLC system equipped with a XEVO TQ-S micro triple quadrupole mass spectrometer (Waters Corp., Milford, MA, USA) for separation and detection. MassLynx 4.1 software (Waters Corp., Milford, MA, USA) was used to acquire data and control the system. The analytes were separated on a Waters ACQUITY UPLC BEH HILIC C18 column (2.1 mm × 50 mm, 1.7 *μ*m). The column temperature was 35°C, and the sample injection volume was 5.0 *μ*L. We performed a gradient elution with solutions A (methanol) and B (0.1% (v/v) formic acid in water) as the mobile phases at a flow rate of 0.4 mL/min: 0-1 min, 80% A and 20% B; 1–4 min, 40% A and 60% B; and 4-5 min, 80% A and 20% B.

Mass spectrometry equipped with electrospray ionization (ESI) source in negative mode was used for multiple reaction monitoring (MRM). We performed mass spectroscopic analysis under the following conditions: quantitative ion pair (m/z): 402.97–96.96; qualitative ion pair (m/z): 402.97–96.96, 402.97–241.07; desolvation gas flow: 700 L/h; cone gas flow: 150 L/h; collision energy: 30–34 eV; capillary voltage: 2800 V; nebulizer pressure: 6.5 bar; and desolvation gas temperature: 550°C.

### 2.5. Determination of the Linear Range, Detection Limit, and Quantification Limit

We measured the prepared matrix standard solutions using UHPLC-MS/MS via an established UHPLC-MS/MS method. The concentration gradient had seven concentration points including 0.05, 0.10, 0.20, 0.40, 0.60, 0.80, and 1.00 g/kg. A standard curve was plotted using the mass concentration of carrageenan (g/kg) as the abscissa (*X*) and the peak area (*μ*S × min) as the ordinate (*Y*). The plot was then used to determine the optimal linear range and correlation coefficient. We used three times the signal-to-noise ratio to calculate the limit of detection (LOD), and 10-fold the signal-to-noise ratio to calculate the limit of quantitation (LOQ) [30].

### 2.6. Determination of Accuracy and Precision of UHPLC-MS/MS

The carrageenan standard stock solutions at three concentrations (0.20 g/kg, 0.40 g/kg, and 0.80 g/kg) were spiked into pork, beef, lamb, chicken, and duck. We performed the pretreatment according to the method described above and measured the recovery of the spiked standard. We measured six replicates of standard samples of the same concentration to calculate the relative standard deviation (RSD) and determine the method's precision.

### 2.7. Application in Real Meat Samples

To validate our strategy, we selected 12 types of commercially available livestock and poultry meat and meat-based products in addition to the five positive samples. We used the methods described above to measure the content of carrageenan in various meat products. The HPLC-MS/MS technique in the Food Supplementary Inspection Method of State Administration for Market Regulation (BJS 201804) was used for verification and to evaluate the practical value of the proposed method [31].

## 3. Results and Discussion

### 3.1. Standard Curve, Linearity, LOD, and LOQ of UHPLC-MS/MS

**Figure 1:**
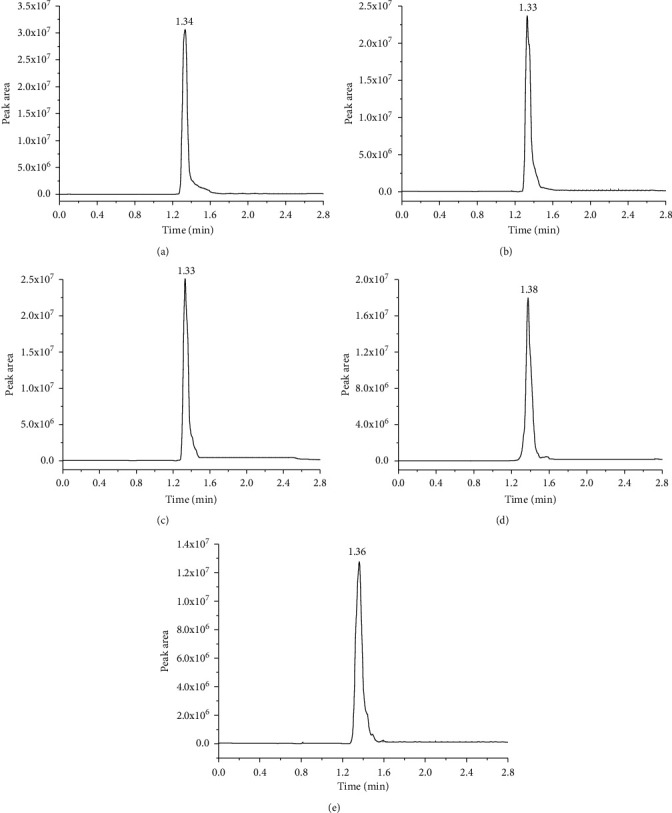
The UHPLC-MS/MS method was developed using the standard chromatograms of carrageenan in five types of livestock and poultry meat samples. The *x*-axis is the detection time (the value marked on the chromatographic peak represents the retention time of carrageenan); the *y*-axis is the peak area. Chromatogram of the carrageenan standard at 0.40 g/kg in (a) pork (retention time 1.34 min), (b) beef (retention time 1.33 min), (c) lamb (retention time 1.33 min), (d) chicken (retention time 1.38 min), and (e) duck (retention time 1.36 min). UHPLC-MS/MS, ultrahigh-performance liquid chromatography-tandem mass spectrometry.

**Figure 2:**
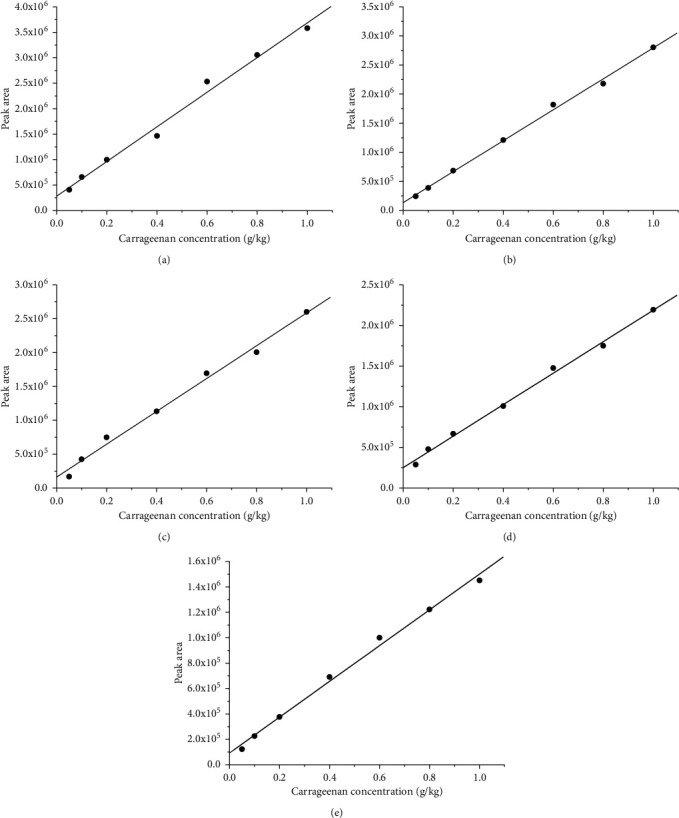
The standard curves based on the UHPLC-MS/MS method for detecting the carrageenan in five different types of livestock and poultry meat (the concentration gradient of carrageenan solution is 0.05, 0.10, 0.20, 0.40, 0.60, 0.80, and 1.00 g/kg, a total of seven concentration points). The *x*-axis is the mass concentration of carrageenan; the *y*-axis is the peak area of the carrageenan standard solution at each concentration. The standard curve of carrageenan in (a) pork matrix, (b) beef matrix, (c) lamb matrix, (d) chicken matrix, and (e) duck matrix. UHPLC-MS/MS, ultrahigh-performance liquid chromatography-tandem mass spectrometry.

We used the UHPLC-MS/MS method to detect the standard working solutions of carrageenan in five types of poultry and meat. The standard curve was plotted to determine the LOQ, the LOD, and the linear range of carrageenan in different matrices. Data from Figures 1 and 2 and Table 1 show that this method efficiently detected carrageenan in livestock and poultry meat and obtained good peak shapes. All samples had a good linear dynamic range of 0.05–1.00 g/kg. The correlation coefficients were greater than 0.99, and the LOD and LOQ were 0.06 g/kg and 0.18 g/kg, respectively, for all samples. Considering the functional characteristics of carrageenan, the amount added in meat products was reported to be 0.25–0.50 g/kg [32]. This study used 0.05 g/kg as the lowest concentration of the standard curve, which met the sensitivity requirements for carrageenan detection. This method offered a shorter detection time, better linear range, and higher sensitivity than previously reported methods [20, 22, 25, 33].

### 3.2. Accuracy and Precision of UHPLC-MS/MS

To verify the accuracy of the method, different concentrations of carrageenan standard were spiked into pork, beef, lamb, chicken, and duck to determine the recovery and precision. We measured each solution concentration in six replicates (Table 2). At the spiked levels, the recovery of carrageenan was 82.06–111.55%, 85.43–112.50%, 89.55–116.00%, 83.80–102.15%, and 82.41–110.90% for pork, beef, lamb, chicken, and duck, respectively; the precision values were 3.27–5.88%, 3.20–5.68%, 4.02–6.84%, 3.61–7.51%, and 0.84–6.11%, respectively. These results indicated that the method has high recovery and good precision and can offer accurate measurements of carrageenan content in complex meat matrices.

### 3.3. Determination of Real Meat Samples

To examine the applicability of the established UHPLC-MS/MS, we next used 12 types of commercially available livestock and poultry meat and meat-based products in addition to the positive controls to measure the carrageenan content. The results are given in Table 3. Carrageenan was detected in only two kinds of restructured steaks; the content was 0.28 g/kg and 0.41 g/kg, respectively. The thin-cut sirloin steak had a clear label of carrageenan addition, but the thick-cut sirloin steak did not. Carrageenan was not detected in the other tested products except for the positive samples. The results were compared with HPLC-MS/MS, and there was no statistical significance between the two methods (*P* > 0.05). The results showed that the method was suitable for the rapid detection of carrageenan content in livestock and poultry meat products and could be used to verify the labeling of food additives and monitor the quality and safety of meat and meat-based products.

## 4. Conclusions

Carrageenan adulteration has become a problem in meat and meat-based products. The addition of carrageenan to livestock and poultry meat may cause contamination risks for heavy metals, foreign substances, and pathogenic microorganisms in meat and meat-based products. Moreover, it adversely affects the health and safety of consumers. Therefore, it is of great significance to develop a rapid detection method for carrageenan content in meat and meat-based products. Here, we developed a UHPLC-MS/MS method to measure the content of carrageenan in livestock and poultry meat. The LOD was 0.06 g/kg, the LOQ was 0.18 g/kg, the recovery of the spiked standard was 82.06–116.00%, and the RSD was lower than 7.51%. These results indicated that the method has a short analysis time, high recovery, good precision, and high repeatability. Furthermore, this method could rapidly measure carrageenan content in livestock and poultry meat and meat-based products.

## Figures and Tables

**Table 1:** Linear equation, correlation coefficient, detection limit, and quantification limit of carrageenan as determined by UHPLC-MS/MS (*n* = 6).

Matrix	Linear equation	Correlation coefficient (*R*^2^)	Linear range (g/kg)	LOD (g/kg)	LOQ (g/kg)
Pork	*Y* = 3398780*X* + 287068.44	0.995	0.05–1.00	0.06	0.18
Beef	*Y* = 2657540*X* + 136688.22	0.999	0.05–1.00	0.06	0.18
Lamb	*Y* = 2422610*X* + 162849.48	0.996	0.05–1.00	0.06	0.18
Chicken	*Y* = 1936610*X* + 251091.83	0.998	0.05–1.00	0.06	0.18
Duck	*Y* = 1408280*X* + 93223.84	0.997	0.05–1.00	0.06	0.18

LOD: limit of detection; LOQ: limit of quantitation; UHPLC-MS/MS: ultrahigh-performance liquid chromatography-tandem mass spectrometry.

**Table 2:** The recovery and precision of spiked carrageenan standard in livestock and poultry meat (*n* = 6).

Matrix	Spiked standard (g/kg)	Measured value (g/kg)	Recovery (%)	RSD (%)
*Pork*	0.20	0.21 ± 0.02	104.75	5.88
	0.40	0.45 ± 0.03	111.55	3.27
	0.80	0.66 ± 0.07	82.06	5.24

*Beef*	0.20	0.23 ± 0.01	112.50	5.68
	0.40	0.38 ± 0.02	95.32	3.20
	0.80	0.68 ± 0.05	85.43	4.61

*Lamb*	0.20	0.23 ± 0.01	116.00	4.02
	0.40	0.36 ± 0.03	89.55	5.40
	0.80	0.84 ± 0.06	105.25	6.84

*Chicken*	0.20	0.20 ± 0.01	102.15	5.53
	0.40	0.39 ± 0.04	96.95	7.51
	0.80	0.67 ± 0.04	83.80	3.61

*Duck*	0.20	0.22 ± 0.01	110.90	0.84
	0.40	0.41 ± 0.02	101.73	6.11
	0.80	0.66 ± 0.01	82.41	2.15

RSD: relative standard deviation.

**Table 3:** Analysis of carrageenan in commercial livestock and poultry meat and meat-based products (*n* = 6).

Sample	Place of production	Carrageenan found (g/kg)	
		UHPLC-MS/MS	HPLC-MS/MS
Pork	Jiangsu, China	Nd	Nd
Beef	Jiangsu, China	Nd	Nd
Lamb	Jiangsu, China	Nd	Nd
Chicken	Jiangsu, China	Nd	Nd
Duck	Jiangsu, China	Nd	Nd
Thick-cut sirloin steak	Zhejiang, China	0.28 ± 0.04	0.29 ± 0.05
Thin-cut sirloin steak	Zhejiang, China	0.41 ± 0.07	0.42 ± 0.09
Prime rib steak	Shanghai, China	Nd	Nd
Charcoal braised pork chop	Beijing, China	Nd	Nd
Bacon	Hubei, China	Nd	Nd
Smoked chicken breast	Shandong, China	Nd	Nd
Chicken breast	Hebei, China	Nd	Nd
A	Jiangsu, China	0.21 ± 0.02	0.22 ± 0.01
B	Jiangsu, China	0.23 ± 0.01	0.22 ± 0.03
C	Jiangsu, China	0.23 ± 0.01	0.24 ± 0.02
D	Jiangsu, China	0.20 ± 0.01	0.21 ± 0.01
E	Jiangsu, China	0.22 ± 0.01	0.23 ± 0.01

Nd: not detected.

## Data Availability

The original data used to support the findings of this study can be obtained from the corresponding author upon request.
